# Polymorphisms of *FCRL3* in a Chinese population with Vogt-Koyanagi-Harada (VKH) syndrome

**Published:** 2009-05-11

**Authors:** Ke Li, Peizeng Yang, Min Zhao, Shengping Hou, Liping Du, Hongyan Zhou, Aize Kijlstra

**Affiliations:** 1 The First Affiliated Hospital of Chongqing Medical University, Chongqing, P.R. China; 2 Zhongshan Ophthalmic Center, Sun Yat-sen University, Guangzhou, P.R. China; 3 Eye Research Institute Maastricht, Department of Ophthalmology, University Hospital Maastricht, Maastricht, the Netherlands

## Abstract

**Purpose:**

The polymorphisms of the Fc receptor-like 3 gene (*FCRL3*), a novel immunoregulatory gene, have been shown to be associated with certain autoimmune diseases. This study was designed to examine whether the polymorphisms of *FCRL3* are associated with susceptibility to Vogt-Koyanagi-Harada (VKH) syndrome in a Chinese population.

**Methods:**

A case-control study was performed in 230 Chinese VKH patients and 301 healthy controls. Four single nucleotide polymorphisms (SNPs; −169C/T, −110A/G, +358C/G, and +1381A/G) in *FCRL3* were detected using polymerase chain reaction restriction fragment length polymorphism (PCR-RFLP). human leukocyte antigen -DR4 (*HLA-DR4*) and *HLA-DRw53* genotyping was performed using PCR techniques.

**Results:**

The results showed that the frequency of haplotype CACG was significantly lower in patients when compared with that in controls (p=0.0018, corrected p [pc]=0.0288). A significantly higher frequency was found for haplotype CGGG in *HLA-DR4* negative patients than in *HLA-DR4* negative controls (p=9.94×10^−8^, Pc=1.59×10^−6^). There were no significant differences in the allele and genotype frequencies of the four investigated SNPs between VKH patients and controls. *HLA-DR4* and *HLA-DRw53* were significantly associated with VKH syndrome (p=3.21×10^−16^ and p=7.08×10^−5^, respectively). Stratification analysis according to *HLA-DR4* and *HLA-DRw53* did not show any association of *FCRL3* polymorphisms with VKH syndrome.

**Conclusions:**

Our study confirms the previous association of *HLA-DR4* and *HLA-DRw53* with VKH syndrome but fails to demonstrate an association between *FCRL3* polymorphisms and VKH syndrome. Haplotype CACG might be a protective haplotype for VKH syndrome, and haplotype CGGG may be a risk haplotype in *HLA-DR4* negative individuals.

## Introduction

Vogt-Koyanagi-Harada (VKH) syndrome is one of the most common uveitis entities in China [1]. The major clinical manifestations of VKH syndrome include panuveitis, pleocytosis in the cerebrospinal fluid, dysacusis, alopecia, poliosis, and vitiligo [2-4]. Although the pathogenesis of VKH syndrome remains uncertain, accumulating evidence suggests that both autoimmune and genetic factors are involved in the development of this disease. Previous research showed that VKH is a T-cell-mediated autoimmune disorder predominantly against melanocytes [5] and that the tyrosinase family proteins may play an important role in VKH disease. Lymphocytes of VKH patients were shown to proliferate in response to tyrosinase or tyrosinase related protein [6]. VKH occurs most commonly in colored people such as certain Asian, American-Indian, and Spanish populations [7], particularly in those carrying the genes coding for the human leukocyte antigen (*HLA*), *HLA-DR4* and *HLA-DRw53* [8-10]. A study in our laboratory recently reported that the frequencies of *HLA-DR4* and *HLA-DRw53* were both significantly increased in VKH patients (odds ratios [OR]=13.74 and OR=4.13, p=3.21×10^–16^ and p=7.08×10^–5^, respectively) [11]. However, the association between VKH syndrome and the *HLA* system does not completely explain the genetic risk for this disease. Investigation of non-*HLA* susceptibility genes for VKH has been an ongoing research subject during recent years [12].

Fc receptor-like genes (*FCRL*s), also known as *FCRH*s (Fc receptor homology), cluster in the midst of the classical Fc receptor genes in the human chromosome 1q21–23 region [13]. *FCRL*s with similarity in structure and sequence to the classical *Fcγ* receptor genes (*FcγR*) contain six immunoglobulin (Ig) superfamily members known as *FCRL1*–*FCLR6* according to their chromosomal order [14]. *FCRL3* is predominantly expressed in lymphoid organs, more precisely in germinal centers, and has been linked to the maturation of B cells [15]. *FCRL3* may play a role in the differentiation of B cells into autoreactive cells and has been presumed to function through modulating signal transduction via activation/inactivation of signaling tyrosine protein kinases [16]. Recently, polymorphisms of *FCRL3* have been reported to be associated with rheumatoid arthritis (RA), systemic lupus erythematosus (SLE) [17], Behcet’s disease [18], autoimmune thyroid disease (AITD) [17,19], and multiple sclerosis (MS) [20,21]. The first two have a strong autoantibody component whereas the latter three are predominately mediated by T-cell response. As an autoimmune disease, VKH may share a common pathogenesis with these autoimmune diseases. Therefore, *FCRL3* was chosen as a target gene for VKH syndrome. Whether SNPs of *FCRL3* are also associated with the susceptibility to VKH syndrome is not yet known, and this question was therefore the subject of the study described here.

## Methods

### Study participants

Patients and controls consisted of 230 Chinese VKH patients and 301 healthy controls. All control subjects were matched ethnically and geographically with the patients. The test subjects were recruited from the Zhongshan Ophthalmic Center of Sun Yat-sen University (Guangzhou, China) and the First Affiliated Hospital of Chongqing Medical University (Chongqing, China). To exclude the immunogenetic backgrounds of different populations, we strictly chose the cases from Chinese Han descendents.

The diagnosis of VKH syndrome followed the revised criteria for this disease [22]. The clinical characteristics of the patients are presented in Table 1. All subjects gave their written informed consent for this study, and the study protocol was approved by the local institutional ethics committee.

**Table 1:** *HLA-DR4* and *HLA-DRw53* distribution and clinical characteristics of 230 patients with VKH syndrome.

**Characteristics**	**VKH Patients**			
	***HLA-DR4*+ n=178 (77.4%)**	***HLA-DR4*– n=52 (22.6%)**	***HLA-DRw53*+ n=202 (87.9%)**	***HLA-DRw5*– n=28 (12.1%)**
Male	103 (80.5%)	25 (19.5%)	116 (90.6%)	12 (9.4%)
Female	75 (73.5%)	27 (26.5%)	86 (84.3%)	16 (15.7%)
Neck stiffness	74 (72.5%)	28 (27.5%)	89 (87.3%)	13 (12.7%)
Alopecia	26 (72.2%)	10 (27.8%)	30 (83.3%)	6 (16.7%)
Poliosis	65 (73.9%)	23 (26.1%)	78 (88.6%)	10 (11.4%)
Vitiligo	41 (74.5%)	14 (25.5%)	48 (87.3%)	7 (12.7%)
Dysacusia	57 (69.5%)	25 (30.5%)	70 (85.4%)	12 (14.6%)
Tinnitus	61 (84.7%)	11 (15.3%)	67 (93.1%)	5 (6.9%)
Scalp hypersensitivity	32 (74.4%)	11 (25.6%)	38 (88.4%)	5 (11.6%)

### Single nucleotide polymorphisms and genotyping

Genomic DNA samples of VKH patients and healthy controls were extracted and isolated from ethylene diamine tetraacetic acid (EDTA)-anticoagulated peripheral blood mononuclear cells (PBMCs) by a conventional salting out method and stored at −70 °C until use.

The four single nucleotide polymorphisms (SNPs) in *FCRL3*, −169C/T (rs7528684 or fcrl3_3), −110G/A (rs11264799 or fcrl3_4), +358C/G (rs945635 or fcrl3_5), and +1381A/G (rs3761959 or fcrl3_6), were genotyped by polymerase chain reaction restriction fragment length polymorphism (PCR-RFLP). Genotyping of the −110G/A SNP was performed according to the method described previously [23]. The primers of the three remaining loci were designed using premier 5.0 software (Premier Biosoft International, Palo Alto, CA). The details of the primers and enzymes used for PCR-RFLP genotyping are presented in Table 2.

**Table 2:** Details of the primers, enzymes, and temperature used in PCR-RFLP genotyping.

**SNPs**	**dbSNP ID**	**Forward primer**	**Reverse primer**	**Tm (°C)**	**Restriction enzyme**
−169C/T (fcrl3_3)	rs7528684	GGAAAATAATACA	GGCTTTAAAA	56.9	BsmFI
		AATGTACAGATTA	AACGGTGGTAC		
−110A/G (fcrl3_4)	rs11264799	CTCAATCCCGGT	CTCATAACAAC	56.9	PleI
		AGTGATACA	TTATGTGAGA		
+358C/G (fcrl3_5)	rs945635	TTATAGCCCATCTA	CCGGGATTGAGA	60.3	HaeIII
		CTCACTCAGGATCA	TACAAACAGCATTT		
+1381A/G (fcrl3_6)	rs3761959	TCCGACTTTTTCA	TGATAGCAGCACTA	60.3	MspI
		GTCTCTAGGTTTT	GCTTGGACATTCA		

PCR was performed in 15 μl volumes containing 7.5 μl Premix Taq (Ex Taq Version; TaKaRa Biotechnology Co. Ltd., Dalian, China), 0.5 μl primers (10 μmol/l), and 0.1 μg of genomic DNA. The PCR products were then digested by the proper restriction enzymes, separated by electrophoresis on 2%−3% agarose gel, and stained with GoldView™ (SBS Genetech, Beijing, China). The images were recorded digitally. Approximately 20% of the PCR samples were directly sequenced to confirm the PCR-RFLP results (Invitrogen Biotechnology Co., Guangzhou, China). *HLA-DR4* genotyping was performed using the PCR sequence specific primers (SSP) method [24]. *HLA-DRw53* typing was performed as described previously [25].

### Statistical analysis

Statistical analysis was performed with the SPSS version 12.0 for Windows (SPSS Inc., Chicago, IL). The Hardy–Weinberg equilibrium (HWE) was tested by the χ^2^ test. We evaluated the frequency of alleles and genotypes in this study using the χ^2^ test or Fisher’s exact test and accounting for multiple testing. The haplotype frequency and linkage disequilibrium (LD) of the SNPs were estimated with the Haploview 3.32 program [26,27]. A haplotype frequency less than 0.03 was not analyzed. All the data were corrected by Bonferroni correction.

## Results

The test subjects consisted of 230 VKH patients (aged 33.4±12.6 years) and 301 healthy controls (aged 35.3±11.9 years). Of the 230 patients, 128 (55.7%) were men and 102 (44.3%) were women. No statistical difference was observed between patients and controls in the distribution of age and gender (p=0.43, p=0.82, respectively). All the cases and controls were in Hardy–Weinberg equilibrium (p>0.05). Therefore, we did the following analysis.

Four SNPs (−169C/T, −110A/G, +358C/G, and +1381A/G) in *FCRL3* were determined in all patients and controls. The results showed that a significantly higher frequency of the G allele at the −110 site of *FCRL3* was observed in VKH patients than in controls (p=0.0129), but this significance was lost after the Bonferroni correction (corrected p [Pc]=0.1032; Table 3). No significant difference was observed in the distribution of other tested alleles or genotypes between VKH patients and controls. The frequency of haplotype CACG was signficantly lower in VKH patients (0.3%) than in controls(3.0%; p=0.0018,Pc=0.0288, χ^2^=9.77; Table 4).

**Table 3:** Frequency of different alleles and genotypes in four *FCRL3* SNPs in VKH patients and controls.

**SNPs**	**Patients n=230 (%)**	**Controls n=301 (%)**	**χ^2^**	**p value**	**Pc**	**OR (95% CI)**
**−169C/T**						
CC	81 (35.2)	87 (28.9)	4.7365	0.0936	NS	
CT	82 (35.7)	135 (44.9)	4.7365	0.0936	NS	
TT	67 (29.1)	79 (26.2)	4.7365	0.0936	NS	
**Alleles**						
C	244 (53.0)	309 (51.3)	0.3071	0.5794	NS	1.071 (0.840-1.366)
T	216 (47.0)	293 (48.7)	0.3071	0.5794	NS	
**−110A/G**						
AA	16 (7.0)	46 (15.4)	8.738	0.0127	NS	
AG	81 (35.5)	98 (32.8)	8.738	0.0127	NS	
GG	131 (57.5)	155 (51.8)	8.738	0.0127	NS	
**Alleles**						
A	113 (24.8)	190 (31.8)	6.174	0.0129	NS	0.707 (0.538-0.930)
G	343 (75.2)	408 (68.2)	6.174	0.0129	NS	
**+358C/G**						
CC	75 (32.6)	100 (33.4)	0.619	0.734	NS	
CG	104 (45.2)	141 (47.2)	0.619	0.734	NS	
GG	51 (22.2)	58 (19.4)	0.619	0.734	NS	
**Alleles**						
C	254 (55.2)	341 (55.7)	0.345	0.557	NS	0.929 (0.727-1.187)
G	206(44.8)	257 (43.0)	0.345	0.557	NS	
**+1381A/G**						
AA	135 (64.6)	150 (58.6)	8.279	0.016	NS	
AG	72 (34.4)	91 (35.5)	8.279	0.016	NS	
GG	2 (1.0)	15 (5.9)	8.279	0.016	NS	
**Alleles**						
A	199 (43.3)	260 (56.5)	1.356	0.5078	NS	—
G	256 (42.7)	344 (57.3)	1.356	0.5078	NS	

Pc: Corrected p value; NS: Not significant; OR: odds ratios.

**Table 4:** Frequencies of *FCRL3* haplotypes in VKH patients and controls.

**Haplotypes**	VKH 2n=456 (%)*	Controls 2n=594 (%)*	**χ^2^**	**Fisher’s p**	**Pc**	**OR (95% CI)**
C A C G	1.57 (0.3)	17.95 (3.0)	9.77	0.0018	0.0288	0.1 (0.02-0.59)
C A G A	78.4 (17.2)	112.0 (18.9)	0.21	0.6454	NS	0.9 (0.67-1.28)
C G C G	47.5 (10.4)	38.1 (6.4)	6.34	0.0118	NS	1.8 (1.13-2.76)
C G G A	91.1 (20.0)	116.9 (19.7)	0.14	0.7137	NS	1.1 (0.78-1.45)
T A C G	14.7 (3.2)	44.4 (7.5)	8.12	0.0044	0.0704	0.4 (0.23-0.78)
T G C G	175.4 (38.5)	219.8 (37.0)	0.81	0.3698	NS	1.1 (0.87-1.46)

The asterisk indicates that the frequency is less than 3% in both VKH cases and control.

We further analyzed the relationship between these SNPs and various extraocular clinical findings including neck stiffness, tinnitus, alopecia, poliosis, dysacusia, scalp hypersensitivity, and vitiligo. The detailed clinical findings of the enrolled VKH patients are shown in Table 1. No significant difference was noted between any clinical parameter stated above and the investigated SNPs.

*HLA-DR4* and *HLA-DRw53* were also determined in VKH patients and controls using the PCR-SSP and PCR method [24,25]. The results showed that the frequencies of both *HLA-DR4* and *HLA-DRw53* were significantly higher in VKH patients than in healthy controls (77.4% versus 19.6%, p=7.34×10^−16^ and 87.8% versus 64.1%, p=5.59×10^−10^, respectively). Stratification analysis was then performed according to the status of *HLA-DR4* and *HLA-DRw53*. A significant difference was found in the frequency of haplotype CGGG between *HLA-DR4* negative patients and *HLA-DR4* negative controls (5.8% versus 0%, p=9.94×10^−8^, Pc=1.59×10^−6^; Table 5). The results did not show any association of allele and genotype frequencies of the four SNPs between VKH patients and normal controls following these stratifications.

**Table 5:** Frequencies of *FCRL3* haplotypes in *HLA-DR4* negative VKH patients and *HLA-DR4* negative controls.

**Haplotypes**	VKH 2n=100 (%)*	Controls 2n=478 (%)*	**χ^2^**	**Fisher’s p**	**Pc**	**OR (95% CI)**
C A C G	2.45 (2.5)	16.79 (3.5)	0.26	0.6102	NS	0.7 (0.18-0.74)
C A G A	16.59 (16.6)	86.29 (18.1)	0.074	0.785502	NS	0.9 (0.52-1.65)
C G C G	6.76 (6.8)	27.20 (5.7)	0.21	0.646662	NS	1.2 (0.51-2.94)
C G G A	23.80 (23.8)	94.85 (19.8)	0.987	0.320533	NS	1.3 (0.77-2.19)
T A C G	2.39 (2.4)	35.27 (7.4)	3.274	0.070433	NS	0.3 (0.08-1.18)
T G C G	33.09 (31)	182.16 (38.1)	0.704	0.401591	NS	0.8 (0.51-1.31)
C G G G	5.78 (5.8)	0.00 (0.000)	28.469	9.94e-08	5.19e-006	

The asterisk indicates that the frequency was less than 3% in both VKH case and control.

## Discussion

We analyzed the association of the aforementioned four SNPs and haplotypes of *FCRL3* in the susceptibility to VKH syndrome in a Chinese population. The results showed that the frequency of haplotype CACG was significantly lower in VKH patients and that the frequency of haplotype CGGG was significantly higher in *HLA-DR4* negative VKH patients. The results also confirmed the association of *HLA-DR4* and *HLA-DRw53* with VKH syndrome as expected. Furthermore, previous studies showed strong associations between *FCRL3* polymorphisms and RA in Asian groups but hardly in Caucasians [16] except a study in Spain [28], which showed that *FCRL3* polymorphisms were associated with RA after stratification by nuclear factor kappa B1 (NF-κB1). VKH syndrome is quite common in both Asian and Spanish populations. The association between *FCRL3* and VKH may be of importance in Chinese VKH patients and Hispanic VKH patients.

The test patients were strictly enrolled as VKH syndrome patients according to the revised criteria [22]. As ethnic confounding could influence the results, we selected only Chinese Han descendents as the test subjects. Moreover, the healthy controls in this study were all limited to persons who came from the same places as the patients, which avoided a geographic bias.

As a novel immunoregulatory gene, *FCRL3* has been reported to be positively associated with various autoimmune diseases in different ethnic populations [17,19,21,29,30], though certain results were conflicting [28,31]. A study by Kochi et al. [17] in Japan found that the four SNPs of *FCRL3* (−169C/T, −110A/G, +358C/G, and +1381A/G) were associated with RA and that SNP −169C/T was associated with AITD and SLE. Furthermore, −169C/T SNP has been shown to influence the level of *FCRL3* expression on B cells by altering the binding affinity of NF-κB, which appears to play a major role in the inflammatory response according to recent studies [32]. Therefore, we speculated that polymorphisms within *FCRL3* could also be related to VKH syndrome.

Although no significant differences were found in the frequencies of the four investigated SNPs of *FCRL3* between VKH patients and normal controls, an increase trend was observed in the frequency of the −110G allele in VKH patients. This result is similar to the study we recently reported in Behcet’s disease in which only the *FCRL3* −110G allele was significantly higher in BD patients and no differences were observed in the remaining three tested SNPs [18]. The polymorphisms of *FCRL3* were reported to be associated with RA in Japan [17,30] and MS in Spain [20,21], but the associations could not be replicated completely in other autoimmune diseases [23,33]. In the study of Graves’ disease [19], three of the four SNPs (except −110A/G SNP) were associated with this autoimmune thyroid disease. A study by Japanese investigators [34] also showed that only −110A/G SNP was associated with autoimmune pancreatitis, but contrary to our result, the frequency of −110G allele was decreased in these patients. Similar negative results were obtained for the same four SNPs of *FCRL3* in other studies concerning type 1 autoimmune hepatitis in the Japanese [35] and SLE in the Chinese population [36]. These observations demonstrated that the relationship between polymorphisms of *FCRL3* and autoimmune diseases is more complex than we thought, especially in some multiple autoimmune disorders.

The further haplotype analysis showed that the frequency of haplotype CACG was significantly lower in VKH patients. This suggests that the haplotype CACG might be a protective haplotype for VKH syndrome. However, the frequency of this haplotype in patients and controls is very low (0.3% versus 3%), making it hard to make any firm conclusion on this significance. The present results were different from those found in BD [18] and SLE [37]. The study of BD in our laboratory found that haplotype CGCG and TACG were both associated with BD in which the first one was found to be significantly higher whereas the latter was significantly lower in BD patients as compared with healthy controls. While in the study of SLE in Spanish individuals, only three alleles were examined and the frequency of haplotype CGA was found to be significantly higher in SLE patients than in controls.

VKH occurs most commonly in pigmented individuals and not in whites, but interestingly, the disease is rare in Africans [7]. It seems that the genetic background in these individuals rather than the amount of pigment is the predisposing factor to VKH syndrome. Various studies have reported that *HLA-DR4* and *HLA-DRw53* were significantly associated with VKH syndrome [8-10]. We therefore analyzed the association of the four SNPs with VKH syndrome based on the *HLA-DR4* and *HLA- DRw53* stratification. We found that the association of *HLA-DR4* and *HLA-DRw53* with VKH syndrome was extremely strong, which is consistent with earlier studies from China [8,38], Japan [10], Korea [39], and Hispanic patients living in California [40]. However, stratification analysis according to the status of *HLA-DR4* and *HLA-DRw53* still showed that none of the four SNPs was associated to VKH syndrome. However, the frequency of haplotype CGGG was significantly higher in *HLA-DR4* negative patients than in *HLA-DR4* negative controls. Haplotype CGGG is probably a risk haplotype predisposing to VKH syndrome in *HLA-DR4* negative subgroups. The samples become small after stratification, consisting of 52 *HLA-DR4* negative VKH patients and 242 *HLA-DR4* negative normal controls. The influence of insufficient sample size can not be excluded yet. Various studies showed that VKH shared epitopes with other autoimmune disease-associated HLA alleles [41-43]. *HLA-DQB1*, -*DR1*, and -*DR4* all were susceptible genes for VKH syndrome [44], and importantly, these HLA antigens were also associated with the susceptibility to other autoimmune diseases including type 1 diabetes [45]and Graves' disease [46].

Systemic disease is a prominent feature of VKH syndrome. There is a large variation in systemic clinical manifestations in Chinese VKH patients [4]. Stratification analysis was therefore performed to investigate the association between the polymorphisms of *FCRL3* and clinical manifestations. The results showed that none of these clinical findings, which included neck stiffness, tinnitus, alopecia, poliosis, dysacusia, scalp hypersensitivity, and vitiligo, was significantly associated with the four SNPs. Similarly, no association was found after gender and age stratification.

In conclusion, no significant correlation could be observed between the *FCRL3* polymorphisms and VKH syndrome in the Chinese population. Haplotype CACG might be a protective haplotype for VKH syndrome. Haplotype CGGG is probably a risk haplotype in *HLA-DR4* negative individuals. Similar to previous studies, we found strong associations of *HLA-DR4* and *HLA-DRw53* with VKH syndrome. Further studies of these SNPs and including other variants in the *FCRL3* region, using larger (multi-center) patient numbers, and including various autoimmune diseases should be performed in the future.
